# Editorial: Regulation of stress tolerance in plants by biostimulants

**DOI:** 10.3389/fpls.2026.1976697

**Published:** 2026-09-28

**Authors:** Michael Moustakas, Ilektra Sperdouli, Julietta Moustaka

**Affiliations:** 1 Department of Botany, School of Biology, Aristotle University of Thessaloniki, Thessaloniki, Greece; 2 Institute of Plant Breeding and Genetic Resources, Hellenic Agricultural Organisation–Demeter (ELGO-Demeter), Thessaloniki, Greece

**Keywords:** arbuscular mycorrhizal fungi, environmental stress, growth-promoting rhizobacteria, microbial biostimulant, non-microbial biostimulants, nutrient uptake, oxidative stress, plant resilience

By the end of this century, crop productivity is estimated to decrease by 11–25% due to climate change, which challenges the ability of agricultural systems to provide food for an increasing world population (Zhao et al., 2017; Wing et al., 2021; Zhu et al., 2022; Moustakas, 2025). Among the recent methodologies used in agricultural science to enhance plant tolerance to adverse environmental conditions owing to climate change, the use of biostimulants is increasing rapidly as an emerging technology (Nephali et al., 2020; Del Buono, 2021; Ma et al., 2022; Monteiro et al., 2022; Moustakas et al., 2024; Di Sario et al., 2025; Silva et al., 2025; Moustakas and Moustaka, 2026).

Plant biostimulants represent a rapidly developing group of agricultural products defined as any substance or microorganism designed to enhance nutrient uptake and utilization, improve crop productivity and quality traits, and strengthen plant resilience against environmental stresses (Drobek et al., 2019; Rouphael and Colla, 2020; Bartucca et al., 2022; Loeza-Lara et al., 2024; Moustakas and Moustaka, 2026; Gaveliene and Jurkoniene). The European Union regulation (EU) 2019/1009 groups plant biostimulants into four areas: (1) those that improve nutrient use efficiency by facilitating plants’ use of available nutrients more effectively, (2) those that enhance abiotic stress tolerance by helping resilience to drought, heat, salinity, and more, (3) those that improve quality traits by improving crop characteristics linked to end use, and (4) those that facilitate nutrient availability in the rhizosphere by improving access to nutrients in the soil [EU 2019/1009] (EU, 2019; Rouphael and Colla, 2020). 

The main categories of biostimulants are microbial and non-microbial. Microbial biostimulants, such as plant growth-promoting rhizobacteria (PGPR) and arbuscular mycorrhizal fungi (AMF), are emerging as important biostimulants that enhance crop yield and improve crop quality by boosting photosynthesis and stress resilience (Moustakas et al., 2020; Lephatsi et al., 2021; Kaushal et al., 2023; Chaudhary et al., 2025). Recognized non-microbial biostimulants include humic and fulvic acids, protein hydrolysates (primarily signaling peptides and free amino acids), seaweed and plant extracts, inorganic compounds, chitosan and biopolymers (Bartucca et al., 2022; Loeza-Lara et al., 2024; Moustakas et al., 2024; Win et al., 2025; Jamil et al., 2026; Moustakas and Moustaka, 2026). Methods for biostimulant application include foliar spraying, soil application, and seed treatment. Both microbial and non-microbial plant biostimulants act by modifying plant primary and secondary metabolism (Colla et al., 2015; Rouphael et al., 2015; Rouphael and Colla, 2020).

In this editorial, we summarize the articles published in this Research Topic to inform readers about recent advances in the field and to provide a useful resource for scientists working in related research areas.

## Stress tolerance in plants by non-microbial biostimulants

As climate change exaggerates the frequency, intensity, and duration of drought episodes, the use of plant biostimulants to improve crop production and quality traits under water-deficit conditions has attracted significant attention (Moustakas and Moustaka, 2026; Gaveliene and Jurkoniene; Rangel Ferreira et al.). Water deficit results in reduced water and nutrient uptake by plants, stomatal closure, decreased photosynthetic efficiency, and increased oxidative damage due to the excessive generation of reactive oxygen species (ROS). Consequently, productivity and yield are also reduced (Moustakas et al., 2022; Sperdouli et al., 2023; Moustakas, 2025; Moustakas et al., 2025).

The physiological mechanisms by which biostimulants contribute to improved drought tolerance include the enhanced accumulation of compatible solutes (proline, soluble sugars, and glycine betaine), the upregulation of antioxidant enzymes to scavenge reactive oxygen species (ROS), the improvement of photosynthesis (preventing also premature leaf senescence), the regulation of hormones, and the development of lateral roots and root hairs to absorb deep soil moisture (Rakkammal et al., 2023; Eswaran et al., 2024; Cassel et al., 2025; Gaveliene and Jurkoniene).

Melatonin is regarded as an anti-stress molecule that functions as a natural antioxidant and plays a positive role in a plant’s resilience to both biotic and abiotic stresses (Moustaka et al., 2024). Exogenous melatonin application alleviated cadmium (Cd)-induced toxicity in *Bletilla striata* by reducing oxidative stress and enhancing antioxidant enzyme activities (Niu et al.). Melatonin decreased hydrogen peroxide and malondialdehyde accumulation while promoting the biosynthesis of beneficial compounds under Cd stress (Niu et al.).

Biostimulants produced from plant waste byproducts (red chicory and cauliflower extracts) were used as maize seed priming substances, and their germination behavior under drought stress was evaluated by calculating several parameters (Wazeer et al.). The data obtained revealed that priming treatments improved both germination behavior and drought tolerance and revealed a genotype-dependent response of maize to drought (Wazeer et al.). Foliar applications of the novel biostimulant “SB”, based on a Glicoligno Lipidic Complex, enhanced cotton physiological efficiency, improved antioxidant and photosynthetic performance leading to higher yield formation, under variable field conditions (Moretti et al., 2026). Moosavi-Nezhad and Meng demonstrated that a calcium‐mobilizing chemical biostimulant resulted in tipburn mitigation in hydroponic lettuce, with results analogous to those of vertical airflow fans under summer greenhouse conditions, with no yield consequences at harvest. Thus, this method can be used wherever vertical airflow fans are impractical.

## Stress tolerance in plants by microbial biostimulants

Among microbial biostimulants, the use of plant growth-promoting microorganisms is emerging as a promising strategy to promote plant growth, increase plant tolerance to stress conditions, repair soil health, and simultaneously reduce dependence on agrochemicals (Wankhade et al., 2025; Yusuf et al., 2025; Dos Santos et al., 2026). Plant growth-promoting bacteria (PGPBs), such as *Pseudomonas* and *Bacillus* strains, improve crop resilience and offer a sustainable strategy to mitigate climate-induced stress (Fanai et al., 2026).

PGPBs combined with exogenous proline–indole-3-acetic acid (IAA) mitigated NaCl-induced salinity stress in *Gerbera jamesonii* cultivars (‘Zingana’ and ‘Sene Vidi’) by reducing malondialdehyde (MDA) accumulation, stabilizing chlorophyll pigments, preserving relative water content, boosting ornamental quality, and thus enhancing physiological resilience to salinity (Karagüzel). Co-inoculation with PGPRs (such as *Bacillus subtilis* or *Priestia megaterium*) combined with a foliar magnesium (Mg) spray lowered oxidative stress markers such as hydrogen peroxide, enhanced soybean net photosynthesis, elevated chlorophyll and carotenoid pigments, and maximized overall grain yield under field stress conditions (de Souza Júnior et al.).

*Trichoderma* is a filamentous fungal genus, with some of its strains known to improve plant growth and induce systemic tolerance to environmental stresses (Harman et al., 2004; Hermosa et al., 2013). Ascaso et al. reported the protective effects of *Trichoderma simmonsii* (strain T137) in wheat (*Triticum aestivum*) plants following 10 days without irrigation. The inoculation resulted in a healthier phenotype and lower oxidative damage (reduced levels of hydrogen peroxide and malondialdehyde), thereby reducing wheat’s need to activate stress pathways (downregulating abscisic acid and osmolyte biosynthesis), while, in parallel, upregulating photosynthesis and carbohydrate metabolism (Ascaso et al.).

*Bacillus velezensis* is a beneficial, Gram-positive, spore-forming soil bacterium widely used as PGPR and a biological control agent. It protects crops against fungal and bacterial pathogens, enhances nutrient uptake, and breaks down environmental pollutants (Rabbee et al., 2019). *Fusarium* wilt, caused by *Fusarium oxysporum f*. sp. *cucumerinum*, seriously affects the yield and quality of cucumber, and it is difficult to control (Li et al.). A novel *Bacillus velezensis* strain (LJ-19), isolated from the cucumber rhizosphere, suppressed *Fusarium* wilt in cucumber via direct antagonism and induction of systemic resistance (Li et al.).

Extracts from the *red alga Lithothamnion* sp., obtained from commercial sources, improved the germination percentage of “Alianca” papaya, promoted leaf and stem growth and development, root collar diameter, leaf and stem dry matter, and total dry matter (Pereira Ramos et al.). Tomato cell cultures elicited with 25 mM methyl-β-cyclodextrins and 100 µM methyl jasmonate served as a novel plant biostimulant, enhancing pepper seed germination and seedling vigor under salt stress (Martí-Guillén et al.). Pepper seed priming with this novel biostimulant improved the germination percentage (+25%) and vigor index (+47%), reduced hydrogen peroxide (H_2_O_2_) by 24%, and promoted peroxidase (POX) activity by 35% (Martí-Guillén et al.).

Shukla et al. (2019) described the beneficial effects of *Ascophyllum nodosum*-based biostimulant extracts on plant growth and stress acclimation, and predicted new opportunities for its application in agriculture and horticulture. Foliar applications of *Ascophyllum nodosum*, *Lithothamnium calcareum*, and melatonin on papaya mitigated recurrent water deficit in ‘Aliança’ papaya seedlings by supporting water retention and lowering leaf water potential (Rangel Ferreira et al.). These biostimulants also enhanced root and stem growth, and restored photosynthesis, improving drought resilience (Rangel Ferreira et al.).

In summary, the articles in this Special Issue conclude that plant biostimulants regulate plant stress resilience by improving nutrient use efficiency, enhancing antioxidant capacity, optimizing primary and secondary metabolism, and modulating stress-related phytohormones to resist adverse environmental factors such as drought, salinity, and heavy metal stress. By examining recent advancements and the field-level implementation, this Special Issue highlights the need to expand biostimulant science to fully realize its potential in driving the transition toward sustainable agriculture.
